# Survival nomogram for medulloblastoma and multi-center external validation cohort

**DOI:** 10.3389/fphar.2023.1247812

**Published:** 2023-11-02

**Authors:** Xiang Li, Jian Gong

**Affiliations:** ^1^ Department of Pediatric Neurosurgery, Beijing Tiantan Hospital, Capital Medical University, Beijing, China; ^2^ Beijing Neurosurgical Institute, Capital Medical University, Beijing, China

**Keywords:** medulloblastoma, training cohort, validation cohort, overall survival, nomogram, end results

## Abstract

**Background:** Medulloblastoma (MB) is a highly malignant neuroepithelial tumor occurring in the central nervous system. The objective of this study was to establish an effective prognostic nomogram to predict the overall survival (OS) of MB patients.

**Materials and methods:** The nomogram was developed using data from a retrospective cohort of 280 medulloblastoma patients (aged 3–18 years) identified from Beijing Tiantan Hospital between 2016 and 2021 as the training cohort. To validate the performance of the nomogram, collaborations were formed with eight leading pediatric oncology centers across different regions of China. A total of 162 medulloblastoma patients meeting the inclusion criteria were enrolled from these collaborating centers. Cox regression analysis, best subsets regression, and Lasso regression were employed to select independent prognostic factors. The nomogram’s prognostic effectiveness for overall survival was assessed using the concordance index, receiver operating characteristic curve, and calibration curve.

**Results:** In the training cohort, the selected variables through COX regression, best subsets regression, and Lasso regression, along with their clinical significance, included age, molecular subtype, histological type, radiotherapy, chemotherapy, metastasis, and hydrocephalus. The internally and externally validated C-indexes were 0.907 and 0.793, respectively. Calibration curves demonstrated the precise prediction of 1-, 3-, and 5-year OS for MB patients using the nomogram.

**Conclusion:** This study developed a nomogram that incorporates clinical and molecular factors to predict OS prognosis in medulloblastoma patients. The nomogram exhibited improved predictive accuracy compared to previous studies and demonstrated good performance in the external validation cohort. By considering multiple factors, clinicians can utilize this nomogram as a valuable tool for individualized prognosis prediction and treatment decision-making in medulloblastoma patients.

## 1 Introduction

Medulloblastoma, a malignant brain tumor primarily affecting children, remains a significant challenge in pediatric oncology (Gajjar and Robinson, 2014). Despite advancements in treatment modalities, including surgery, radiation therapy, and chemotherapy, the overall prognosis for patients with medulloblastoma varies widely due to tumor heterogeneity and the complex interplay of genetic and clinical factors (Bouffet, 2021). Consequently, accurate prediction of individual patient outcomes is essential for tailoring treatment strategies and improving survival rates. In recent years, the development of prognostics has emerged as a valuable tool in oncology (Iasonos et al., 2008). These predictive models combine various clinical and pathological variables to estimate the likelihood of specific outcomes for patients. Nomograms provide clinicians with a visual representation of the probability of survival or recurrence, facilitating personalized treatment decisions and enhancing patient care.

Building upon this approach, a group of researchers has recently developed a novel Survival Nomogram specifically tailored for medulloblastoma patients (Dasgupta et al., 2019; Zhu et al., 2020; Liu and Sun, 2022). The nomogram incorporates crucial prognostic factors, such as age at diagnosis, histological subtype, resection extent, and molecular subgroup classification. Recent research has found that pathological classification is not strongly correlated with clinical prognosis (Entz-Werle et al., 2008). For medulloblastoma, molecular classification plays a significant role in determining patient outcomes (Northcott et al., 2019). However, studies are scarce that incorporate molecular subtyping into prognostic models. This study introduces a novel approach by integrating molecular subtyping into a clinical prognostic model. By integrating these variables, the nomogram generates a risk score that enables accurate predictions of individual patient survival probabilities.

To validate the efficacy and reliability of the Survival Nomogram, the research team has embarked on a groundbreaking multi-center external validation study. Collaborating with leading pediatric oncology centers across different geographical regions, the study aims to assess the nomogram’s performance using an independent cohort of medulloblastoma patients. This approach ensures the generalizability and robustness of the nomogram in diverse clinical settings, enhancing its potential as a practical tool for oncologists worldwide. The multi-center external validation cohort consists of a large sample of medulloblastoma patients spanning various demographics, treatment protocols, and follow-up durations. By comparing the predicted survival probabilities generated by the nomogram with the observed patient outcomes, the researchers will evaluate the nomogram’s accuracy, discrimination, and calibration. These analyses will confirm the nomogram’s validity and provide insights into its potential limitations and areas for further refinement.

The implications of a validated Survival Nomogram for medulloblastoma are profound. By enabling accurate individualized predictions, this predictive model can guide treatment decisions, helping clinicians strike a delicate balance between aggressive interventions and minimizing long-term treatment-related complications. Moreover, the nomogram promises to optimize clinical trial designs, stratify patients for targeted therapies, and facilitate long-term survivorship planning.

In conclusion, the development and validation of a Survival Nomogram for medulloblastoma represent a significant advancement in pediatric oncology. By harnessing the power of prognostic modeling, this nomogram offers a personalized approach to patient management and prognosis prediction. The ongoing multi-center external validation study aims to provide robust evidence supporting the nomogram’s clinical utility and establish it as a valuable tool in the battle against medulloblastoma.

## 2 Patients and methods

### 2.1 Study design

This study was designed to develop a Survival Nomogram for medulloblastoma patients and validate its performance using a multi-center external validation cohort.

#### 2.1.1 Nomogram development

a. Patient Selection: A retrospective cohort of 280 medulloblastoma patients (Ages 3–18 years) was identified from Beijing Tiantan Hospital between 2016 and 2021. Only patients with confirmed medulloblastoma diagnosis and complete clinical and molecular data were included. b. Data Collection: Relevant clinical information such as age at diagnosis, histological subtype, metastasis, tumor texture, hydrocephalus, resection extent, and adjuvant therapy details were collected from patient medical records. Genetic profiling obtained molecular subtyping information, including WNT, SHH, Group 3, and Group 4. c. We have employed three methods for variable selection. Method 1: Univariable Cox and multivariable Cox analysis regression analysis were conducted to screen for potential prognostic variables. Each variable was assessed individually for its association with the survival outcome. Method 2: Best subsets regression (BSR) was performed to screen for variables that showed significant associations with the survival outcome. Different combinations of variables were evaluated, starting with subsets containing only one variable and gradually increasing the size of the subsets. The BSR was used to determine the optimal variable combination by maximizing the adjusted *R* ^2^ value. Method 3: Lasso regression, combined with cross-validation, was employed in the variable selection process. This method utilized regularization techniques to shrink the coefficients of irrelevant variables and select the most relevant ones. Lasso regression with cross-validation was used to determine the variable combination by selecting the λ value that corresponds to the minimum mean squared error (MSE).

#### 2.1.2 Final variable selection

After constructing three models through variable selection using the methodologies, we proceeded with a comprehensive analysis. Consequently, a final model, referred to as the “Summary model,” was formulated. To determine the most optimal model, we conducted a rigorous comparison of the Akaike information criterion (AIC) and area under the curve (AUC) values among the four models (COX, BSR, Lasso, Summary). This meticulous evaluation allowed us to identify the model with superior predictive performance and robustness, culminating in the selection of the most suitable and reliable model for our study.

#### 2.1.3 External validation cohort

a. Collaboration with Multiple Centers: To validate the nomogram’s performance, collaborations were established with eight leading pediatric oncology centers across different regions. b. Patient Enrollment: A total of 162 medulloblastoma patients who met the inclusion criteria were enrolled from the collaborating centers. Detailed clinical and molecular data were collected, including age at diagnosis, histological subtype, metastasis, tumor texture, hydrocephalus, extent of resection, and adjuvant therapy and molecular subtyping. c. Comparative Analysis: The developed nomogram was applied to the external validation cohort. The predicted survival probabilities generated by the nomogram were compared with the observed survival outcomes of the validation cohort. Calibration plots and Harrell’s concordance index were performed to assess the nomogram’s accuracy and discrimination in the independent patient population. d. The final model compared with the model built using only clinical factors.

Ethical approvals were obtained from the institutional review boards of all participating centers, ensuring patient privacy and data protection. Informed consent was obtained from patients or their legal guardians.

#### 2.1.4 Statistical analyses

Statistical analyses were performed using R software, and the nomogram was constructed using the “rms” package in R software (version 4.2.3). In addition, to build the model in R language, we also used the following packages: “survival,” “plyr,” “MASS,” “leaps,” “glmnet,” “riskRegression,” “ggplot2,” “pec” and “ggDCA”. A novel nomogram including all the independent prognostic factors was developed to predict 1-, 3- and 5-year OS for medulloblastoma patients. Statistical analysis categorical variables are expressed as percentages and continuous variables as the mean ± standard deviation (SD). All *p*-values were two-sided, and *p* < 0.05 was considered statistically significant.

## 3 Results

### 3.1 Patient baseline characteristics

A total of 280 eligible patients with medulloblastoma were enrolled from Beijing Tiantan Hospital as the training cohort. They had an average age of 7.74 years (SD 3.25). Classic histology was the most common subtype at 68.57%, followed by desmoplastic/nodular at 23.21%, and large cell/anaplastic at 2.86%. Metastasis was present in 17.50% at diagnosis. Hydrocephalus occurred in 88.21% of patients. Gross total resection was achieved in 43.93%, while 56.07% had subtotal resection. Most patients received adjuvant therapy, including chemotherapy (89.29%) and radiotherapy (95.36%). Molecular subtyping revealed Group 3 10.36% and Group 4 50.00% in training cohort, WNT in 14.64%, and SHH-activated subtypes in 25.00%. Apart from molecular subtyping, there were no significant differences observed between males and females in the other variables (chi-square tests). Additionally, 162 cases of patients with medulloblastoma from eight other centers were selected and utilized as the external validation cohort. In the external validation cohort, the patients had an average age of 8.24 years (standard deviation 3.51). Classic histology was the most common subtype, accounting for 77.16%, followed by desmoplastic/nodular at 14.81%, and large cell/anaplastic at 3.70%. Metastasis was present in 14.81% at the time of diagnosis. Hydrocephalus was observed in 76.54% of the patients. Gross total resection was achieved in 80.25% of cases, while 19.75% underwent subtotal resection. The majority of patients received adjuvant therapy, including chemotherapy (81.48%) and radiotherapy (87.65%). Molecular subtyping revealed that 12.35% belonged to Group 3 and 42.59% to Group 4 in the training cohort, 17.90% were classified as WNT subtype, and 27.16% as SHH-activated subtypes. The clinicopathological characteristics of the patients are summarized in Table 1. Some of the risk factors of medulloblastoma previous referenced (Rieken et al., 2011; Guo et al., 2020; Zhu et al., 2020; Liu and Sun, 2022) research utilized included age, gender, tumor size, histological type, extent of surgical resection, radiation therapy, chemotherapy, and metastasis. In our study, we added some significant important variables, including hydrocephalus, tumor texture, and most importantly, molecular subtyping.

**TABLE 1:** Patient baseline characteristics.

Characteristics	Training cohort				External validation cohort			
	Male (*N* = 184)	Female (*N* = 96)	Total (*N* = 280)	*p*-value	Male (*N* = 105)	Female (*N* = 57)	Total (*N* = 162)	*p*-value
Age								
Mean ± SD	7.65 ± 3.27	7.91 ± 3.23	7.74 ± 3.25		8.22 ± 3.44	8.28 ± 3.67	8.24 ± 3.51	
Median [min-max]	7.00 [3.00, 18.00]	7.00 [3.00, 15.00]	7.00 [3.00, 18.00]		7.00 [3.00, 17.00]	8.00 [3.00, 17.00]	8.00 [3.00, 17.00]	
Molecular				3.40E−05				0.96
G3	21 (7.50%)	8 (2.86%)	29 (10.36%)		13 (8.02%)	7 (4.32%)	20 (12.35%)	
G4	104 (37.14%)	36 (12.86%)	140 (50.00%)		44 (27.16%)	25 (15.43%)	69 (42.59%)	
SHH	45 (16.07%)	25 (8.93%)	70 (25.00%)		28 (17.28%)	16 (9.88%)	44 (27.16%)	
WNT	14 (5.00%)	27 (9.64%)	41 (14.64%)		20 (12.35%)	9 (5.56%)	29 (17.90%)	
Histological				0.54				0.4
Classic	122 (43.57%)	70 (25.00%)	192 (68.57%)		83 (51.23%)	42 (25.93%)	125 (77.16%)	
Desmoplastic	44 (15.71%)	21 (7.50%)	65 (23.21%)		16 (9.88%)	8 (4.94%)	24 (14.81%)	
Large cell/anapla-stic histology	6 (2.14%)	2 (0.71%)	8 (2.86%)		2 (1.23%)	4 (2.47%)	6 (3.70%)	
Medulloblastoma with extensive nodularity	12 (4.29%)	3 (1.07%)	15 (5.36%)		4 (2.47%)	3 (1.85%)	7 (4.32%)	
Radiotherapy				0.98				0.79
No	8 (2.86%)	5 (1.79%)	13 (4.64%)		14 (8.64%)	6 (3.70%)	20 (12.35%)	
Yes	176 (62.86%)	91 (32.50%)	267 (95.36%)		91 (56.17%)	51 (31.48%)	142 (87.65%)	
Chemotherapy				0.37				0.38
No	17 (6.07%)	13 (4.64%)	30 (10.71%)		22 (13.58%)	8 (4.94%)	30 (18.52%)	
Yes	167 (59.64%)	83 (29.64%)	250 (89.29%)		83 (51.23%)	49 (30.25%)	132 (81.48%)	
Metastasis				0.67				0.62
No	150 (53.57%)	81 (28.93%)	231 (82.50%)		91 (56.17%)	47 (29.01%)	138 (85.19%)	
Yes	34 (12.14%)	15 (5.36%)	49 (17.50%)		14 (8.64%)	10 (6.17%)	24 (14.81%)	
Resection				0.55				0.92
GTR	78 (27.86%)	45 (16.07%)	123 (43.93%)		85 (52.47%)	45 (27.78%)	130 (80.25%)	
STR	106 (37.86%)	51 (18.21%)	157 (56.07%)		20 (12.35%)	12 (7.41%)	32 (19.75%)	
Texture				0.14				0.12
Soft	127 (45.36%)	75 (26.79%)	202 (72.14%)		44 (27.16%)	32 (19.75%)	76 (46.91%)	
Tone	57 (20.36%)	21 (7.50%)	78 (27.86%)		61 (37.65%)	25 (15.43%)	86 (53.09%)	
Hydrocephalus				0.64				0.66
No	20 (7.14%)	13 (4.64%)	33 (11.79%)		23 (14.20%)	15 (9.26%)	38 (23.46%)	
Yes	164 (58.57%)	83 (29.64%)	247 (88.21%)		82 (50.62%)	42 (25.93%)	124 (76.54%)	

GTR, gross total resection; STR, subtotal resection.

### 3.2 The relationship between pathological classification and molecular classification

**FIGURE 1:**
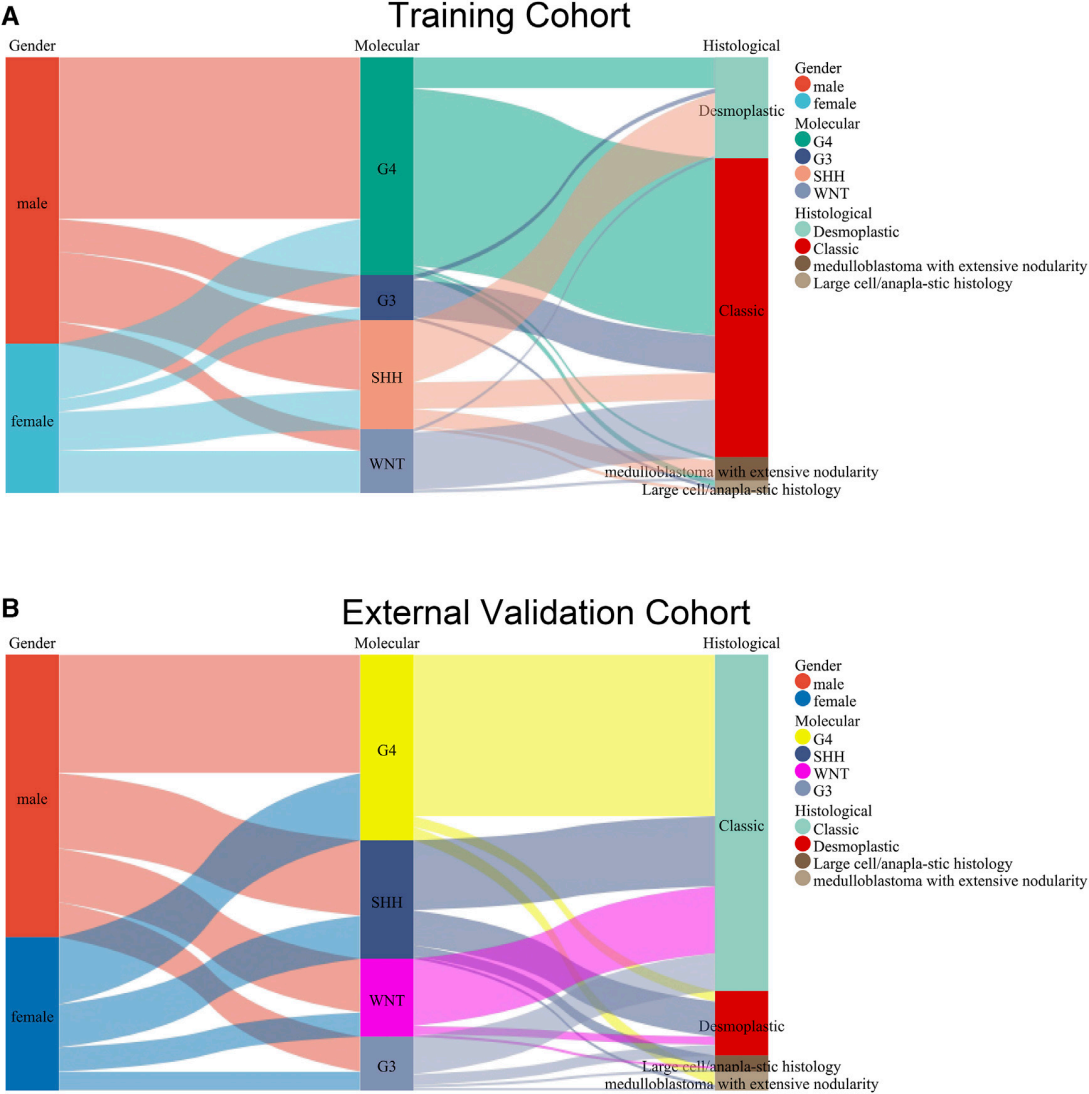
Sankey diagram displaying the relationship between pathological classification and molecular subtyping in the training group **(A)** and the external validation group **(B)**.

The Sankey diagram displays the heterogeneity of pathological classification and molecular subtyping between the training group and the external validation group (Figures 1A, B). Indeed, it is evident that a single pathological classification can correspond to multiple molecular subtypes. Multiple studies have consistently demonstrated a significant correlation between molecular subtypes of medulloblastoma and prognosis (Schwalbe et al., 2017). This represents the complex relationship between pathological classification and molecular classification.

### 3.3 Feature selection and nomogram construction

**FIGURE 2:**
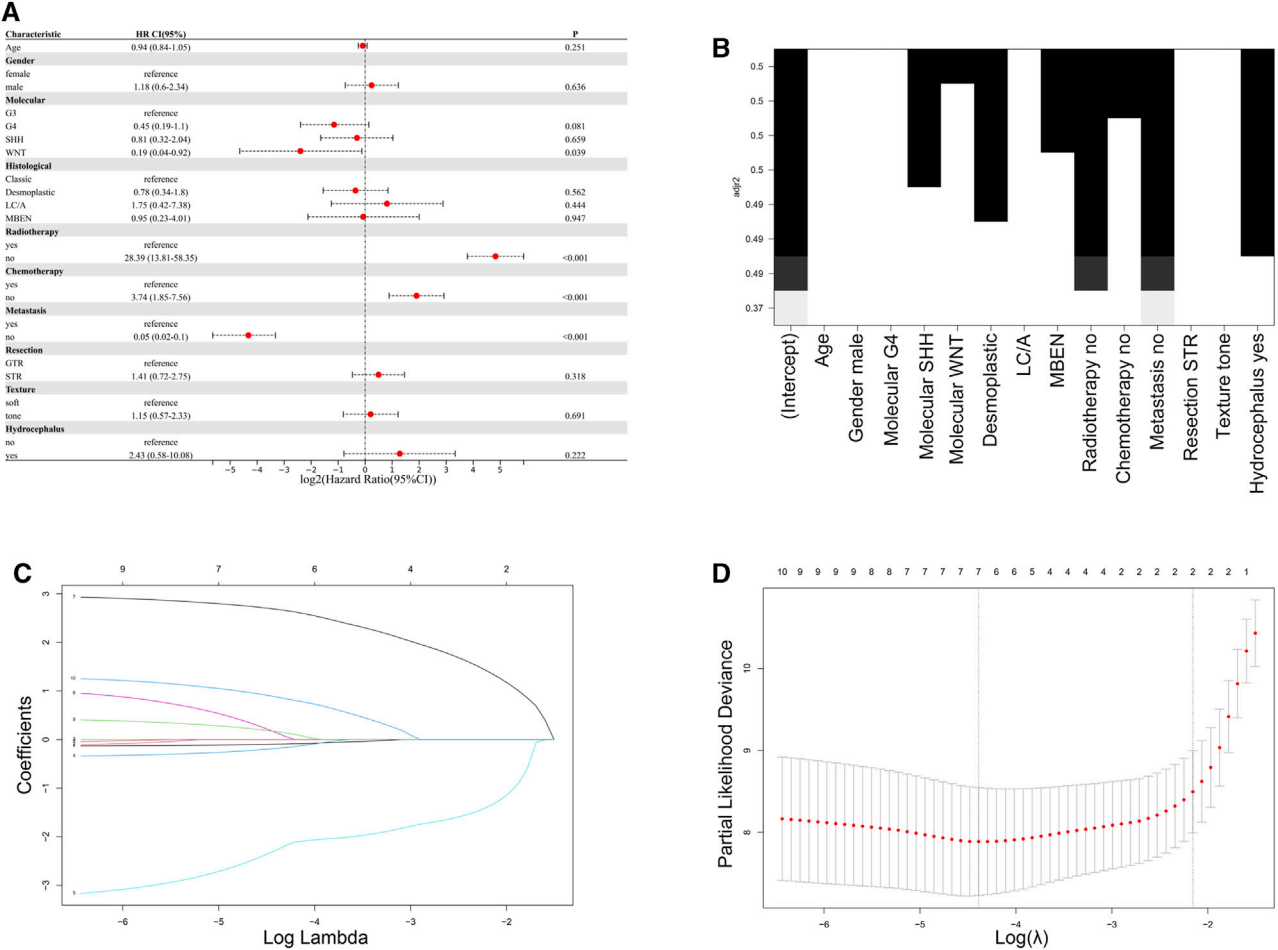
Comparison of feature selection methods for the development of the nomogram. **(A)** Results of univariate Cox regression analysis. **(B)** Results of best subset regression analysis. **(C,D)** LASSO regression variable selection process.

A total of nine clinical parameters were included in the training cohort. **Method 1:** In the univariate Cox regression analysis, Molecular subtype, Radiotherapy, Chemotherapy, and Metastasis were associated with OS (*p* < 0.05) (Figure 2A). And the variables selected were then included in the multivariable Cox analysis. The final Cox model included four variables: Molecular subtype, Radiotherapy, Chemotherapy, and Metastasis. The AIC value for this model was 305.7054. **Method 2:** In the BSR analysis, the goal was to identify the best combination of variables based on evaluation criteria, such as minimizing Mallows’ Cp, maximizing adjusted *R* ^2^, and minimizing the Bayesian information criterion. The analysis aimed to determine the most informative subset of variables for predicting survival in medulloblastoma patients. The results of the best subset regression revealed that a combination of six variables was selected for inclusion in the model. These variables are Molecular subtype, Histological, Radiotherapy, Chemotherapy, Metastasis, and Hydrocephalus. This subset regression analysis considered all possible combinations of variables and evaluated their performance based on adjusted *R* ^2^. The selected combination of variables demonstrated the highest adjusted *R* ^2^ among all evaluated combinations, indicating its strong association with the survival outcome in medulloblastoma patients (Figure 2B). This subset regression analysis provides valuable insights into the significant predictors of survival in medulloblastoma, incorporating both molecular and clinical factors. The AIC value for this model was 298.9553. **Method 3:** LASSO regression is a technique used to address overfitting and severe multicollinearity in regression models by introducing a penalty function that shrinks the regression coefficients of variables. The choice of the λ value determines which variables contribute to an optimal model, and cross-validation is employed to find the best λ value. The λ value corresponding to the minimum mean squared error (MSE) determines the variables included in the model. The graph illustrates the partial-likelihood deviance as a function of log(λ) (Figures 2C, D). The selected variables for the LASSO regression model are Age, Histological, Radiotherapy, Chemotherapy, Metastasis, Tumor Texture, and Hydrocephalus. The AIC value for this model was 303.3649.

**FIGURE 3:**
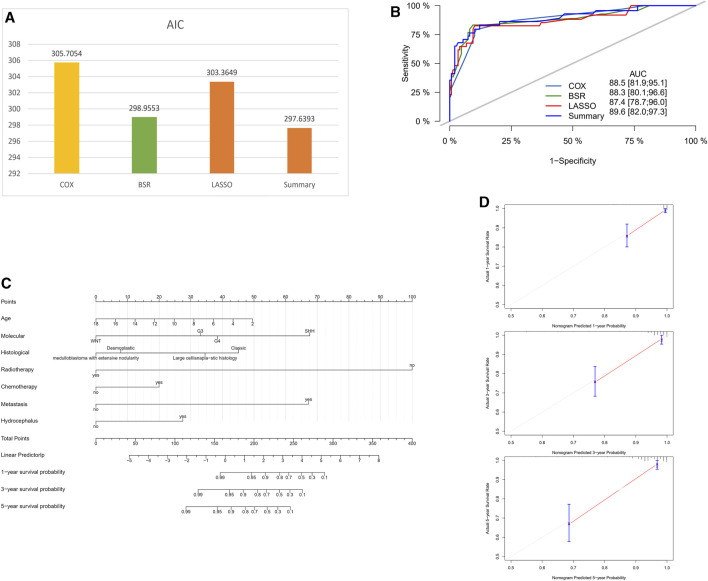
Comparison of different models for nomogram construction. **(A)** Comparison of AIC values for different models. **(B)** Comparison of AUC values for different models. **(C)** Final selection of variables for the summary model. **(D–F)** Calibration curves for the nomogram in the training cohort (1-, 3-, and 5-year OS).

After comparison, it was determined that the model constructed with the variables Age + Molecular + Histological + Radiotherapy + Chemotherapy + Metastasis + Hydrocephalus, obtained through comprehensive analysis (Summary model), performed better. The variable of tumor texture was excluded because adding this variable made only a minimal contribution to the model, and the resulting AIC value was not the lowest. Summary model had the highest AUC value and the lowest AIC value (Figures 3A, B). Therefore, based on the data, the final selection was to construct a nomogram using these seven factors: Age, Molecular subtype, Histological, Radiotherapy, Chemotherapy, Metastasis, and Hydrocephalus (Figure 3C). The calibration curves of the nomogram showed high uniformity between the predicted and actual probabilities of 1-,3- and 5-year OS in the training cohort (Figures 3D–F). The continuous calibration curve also demonstrates the model’s strong predictive capability of summary model (Supplementary Figure S1). The DCA curve of the Summary model mostly lies above the curves of the other three models. This indicates that, at most patient probability thresholds, the Summary model achieves the highest net benefit. Along this curve, the Summary model performs well within the threshold range of 0.3–0.9 and demonstrates a stronger advantage compared to the other three models (Supplementary Figure S2).

### 3.4 Performance and validation of the nomogram

**FIGURE 4:**
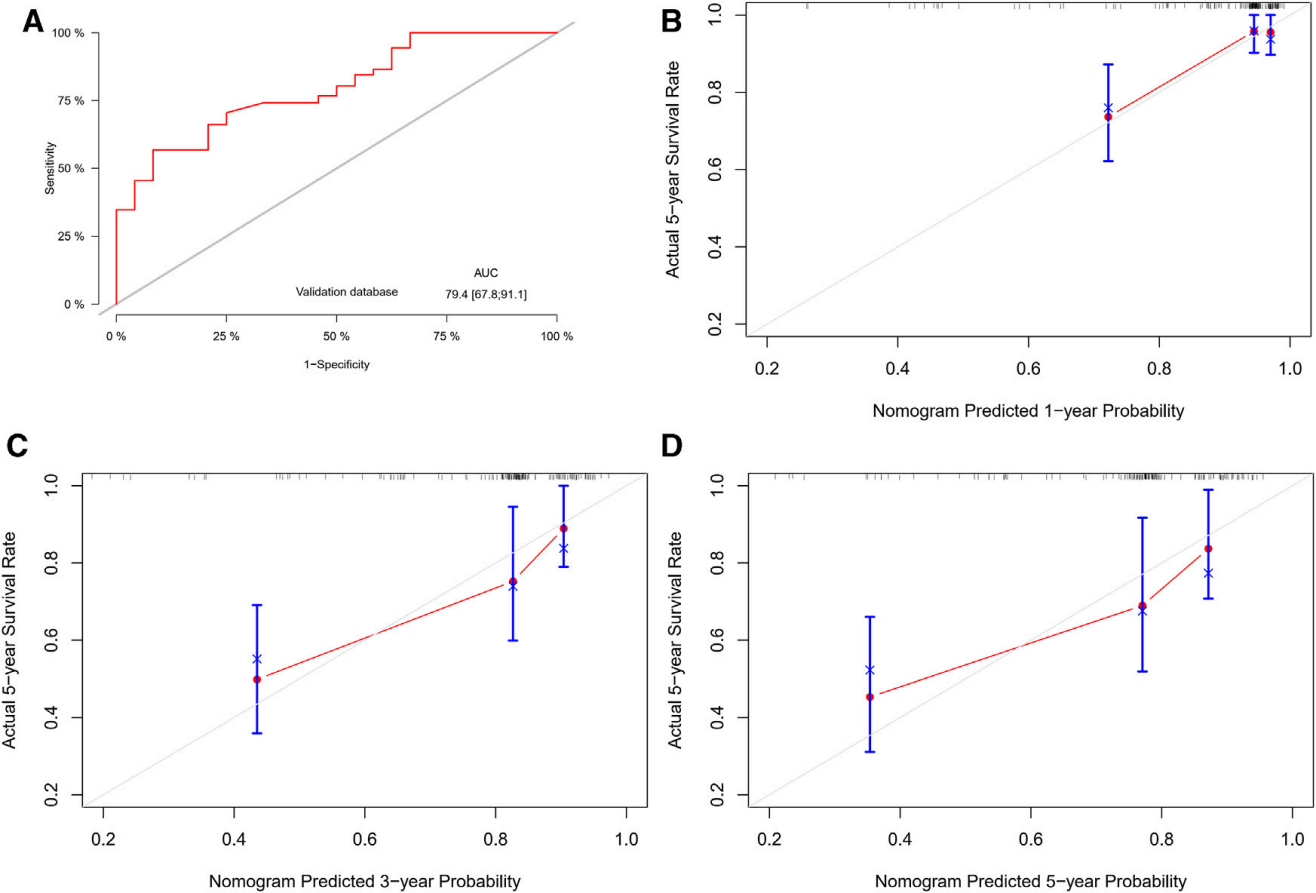
Performance and validation of the nomogram. **(A)** ROC curves for the nomogram in the external validation cohort. **(B–D)** Calibration curves for the nomogram in the external validation cohort (1-, 3-, and 5-year OS).

The C-index values obtained from the nomogram were higher in both the training cohort (0.907) and the external validation cohort (0.793) compared to a previous study (Guo et al., 2020) (training cohort, 0.681; external validation cohort, 0.644). Furthermore, the nomogram demonstrated good performance in predicting the overall survival prognosis in the external validation cohort, as evidenced by the time-dependent ROC curves (Figure 4A). Additionally, the calibration curves of the nomogram exhibited a high level of agreement between the predicted probabilities and the actual probabilities of 1-, 3-, and 5-year OS in the external validation cohort (Figures 4B–D). In the validation set, continuous calibration curves demonstrate the predictive capability of the model (Supplementary Figure S3).

### 3.5 The comparison between the final nomogram and the model using only clinical factors

**FIGURE 5:**
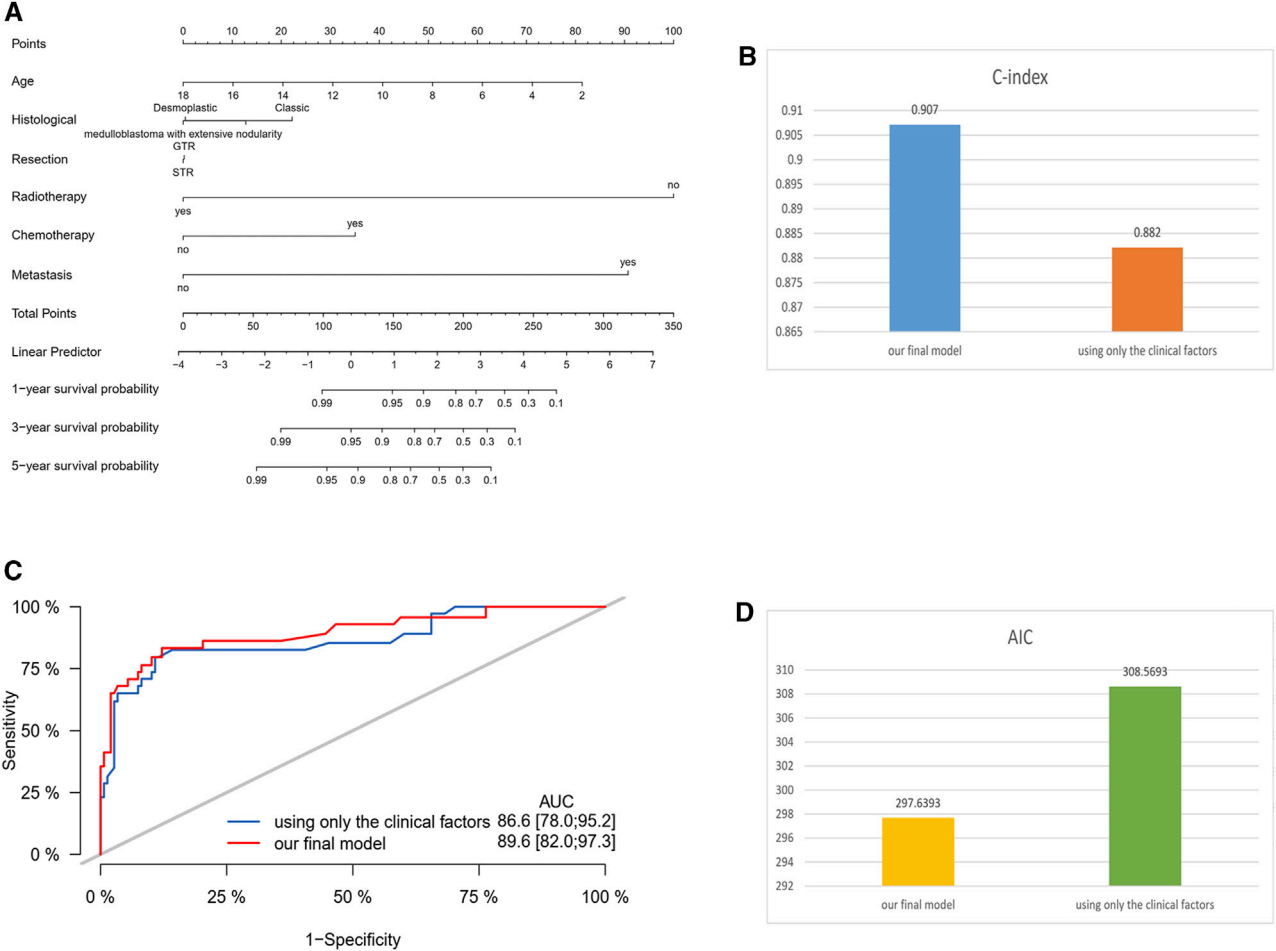
Comparison between the final nomogram and the model using only clinical factors. **(A)** Nomogram of the model using only clinical factors. **(B)** C-index comparison. **(C)** ROC curve comparison. **(D)** AIC value comparison.

Several studies have indicated that age, histological subtype, extent of surgical resection, radiotherapy, chemotherapy, and metastatic status are prognostic risk factors for medulloblastoma (Packer et al., 2006; Rieken et al., 2011; Thompson et al., 2016; Huang et al., 2017; Guo et al., 2020; Zhu et al., 2020; Liu and Sun, 2022). We compared the final model constructed using our own data with the model using only clinical factors. The results showed that the model we constructed had a higher C-index, lower AIC value, and a larger area under the ROC curve (Figures 5A–D).

## 4 Discussion

In this study, we aimed to develop a predictive model for overall survival prognosis in patients with medulloblastoma by incorporating both clinical and molecular factors. Based on their histopathological features, medulloblastomas can be classified into four subtypes: classic medulloblastoma, large cell/anaplastic medulloblastoma, desmoplastic/nodular medulloblastoma, and medulloblastoma with extensive nodularity (Louis et al., 2007). The heterogeneity of pathological classification and molecular subtyping was evident between the training and external validation groups, indicating that the prognosis varied greatly in different cases, emphasizing the importance of incorporating molecular subtyping into predictive models. Previous studies have shown a significant correlation between molecular subtypes of medulloblastoma and prognosis, further justifying the inclusion of this factor in the predictive model (Archer et al., 2017), but currently, there is limited research that incorporates molecular subtyping as a variable in predictive models. We utilized three different methods for variable selection: univariate Cox regression analysis, Best Subsets Regression, and LASSO regression. These three methods can effectively screen out variables that are significantly associated with clinical prognosis (Emura et al., 2019; Kwong et al., 2020; McEligot et al., 2020). By innovatively applying these three methods to screen variables associated with medulloblastoma prognosis, we enhance the accuracy of the final predictive model. After comparing the results, we constructed a nomogram using seven factors: Age, Molecular subtype, Histological, Radiotherapy, Chemotherapy, Metastasis, and Hydrocephalus. The summary model had the highest AUC value and the lowest AIC value. This also confirms the credibility and effectiveness of the model we constructed.

The univariate Cox regression analysis revealed that Molecular subtype, Radiotherapy, Chemotherapy, and Metastasis were associated with OS. BSR identified a combination of six variables: Molecular subtype, Histological, Radiotherapy, Chemotherapy, Metastasis, and Hydrocephalus. LASSO regression, on the other hand, selected Age, Histological, Radiotherapy, Chemotherapy, Metastasis, Tumor texture, and Hydrocephalus. The variables screened through these three methods have been previously validated to be associated with prognosis in existing research on medulloblastoma (Archer et al., 2017; Guo et al., 2020; Aras et al., 2021; Franceschi et al., 2021). This aligns with our study findings. Considering the clinical significance and variables selected by all three methods, a comprehensive approach was adopted, resulting in a model with seven variables. First, different pathology subtypes of MB may indicate distinct biological behaviors that can influence treatment strategies. Large-cell and anaplastic MB are considered high-risk diseases, indicating poorer survival rates, and requiring more aggressive chemotherapy and higher radiation doses. On the other hand, desmoplastic nodular MB may exhibit better outcomes. In our study, Large-cell and anaplastic MB showed the highest risk factor among the histopathological subtypes, which aligns with previous research findings (Huang et al., 2017). Second, Chang staging for MB was introduced in the 1960s, which classified MB patients into M0, M1, M2, M3, or M4 and T1, T2, T3a, T3b, or T4 according to their clinical features. Tumor metastasis indicates a poor prognosis, which is consistent with our research findings (Dufour et al., 2012). Third, for medulloblastoma, postoperative adjuvant therapy is crucial, especially radiation therapy. Previous studies also support our findings (Menyhárt and Győrffy, 2020). It is worth noting that the tumor resection extent did not show a clear correlation with prognosis in our experimental and validation datasets. The prognostic benefit of increased resection extent for patients with medulloblastoma is attenuated after molecular subgroup affiliation is considered. Although maximum safe surgical resection should remain the standard of care, surgical removal of small residual portions of medulloblastoma is not recommended when the likelihood of neurological morbidity is high because there is no definitive benefit to gross total resection compared with near-total resection (Thompson et al., 2016). This suggests that when the tumor adheres to the brainstem or some important neurovascular structure, it is not necessary to aggressively pursue complete tumor resection to avoid catastrophic consequences.

The performance of the nomogram constructed using the seven selected factors was evaluated using the C-index and compared to a previous study (Guo et al., 2020). The C-index values obtained from the nomogram were higher in both the training cohort and the external validation cohort, indicating improved predictive accuracy. The nomogram demonstrated good performance in predicting the OS prognosis, as supported by the ROC and calibration curves in both the training and external validation cohorts.

The incorporation of both clinical and molecular factors in the nomogram provides a more comprehensive and accurate prediction of the OS prognosis in medulloblastoma patients. By considering variables such as age, treatment modalities (radiotherapy and chemotherapy), metastasis status, molecular subtype, histological classification, and the presence of hydrocephalus, clinicians can make more informed decisions regarding patient management and treatment strategies.

It is important to note that this study has certain limitations. The data used for model development and validation were collected from a single institution and eight external centers, which may introduce bias and limit the generalizability of the findings. Further external validation in larger and more diverse patient populations is necessary to validate the performance of the nomogram. Additionally, the study did not consider other potential prognostic factors such as genetic mutations or gene expression profiles, which could further enhance the predictive accuracy of the model.

## 5 Conclusion

In conclusion, this study developed a nomogram incorporating clinical and molecular factors for predicting the OS prognosis in medulloblastoma patients. The nomogram demonstrated improved predictive accuracy compared to a previous study and exhibited good performance in the external validation cohort. By considering multiple factors, clinicians can utilize this nomogram as a valuable tool for individualized prognosis prediction and treatment decision-making in medulloblastoma patients. Further research and validation are warranted to refine and optimize the predictive model.

## Data Availability

The original contributions presented in the study are included in the article/Supplementary Material, further inquiries can be directed to the corresponding author.

## References

[B1] Aras Y. Dölen D. İribas Çelik A. Kılıç G. Kebudi R. Ünverengil G. (2021). Effects of different molecular subtypes and tumor biology on the prognosis of medulloblastoma. Child's Nerv. Syst. 37 (12), 3733–3742. ChNS : official journal of the International Society for Pediatric Neurosurgery. 10.1007/s00381-021-05350-1 34550414

[B2] Archer T. C. Mahoney E. L. Pomeroy S. L. (2017). Medulloblastoma: molecular classification-based personal therapeutics. Neurother. J. Am. Soc. Exp. Neurother. 14 (2), 265–273. 10.1007/s13311-017-0526-y PMC5398996 28386677

[B3] Bouffet E. (2021). Management of high-risk medulloblastoma. Neuro-Chirurgie. 67 (1), 61–68. 10.1016/j.neuchi.2019.05.007 31229532

[B4] Dasgupta A. Gupta T. Pungavkar S. Shirsat N. Epari S. Chinnaswamy G. (2019). Nomograms based on preoperative multiparametric magnetic resonance imaging for prediction of molecular subgrouping in medulloblastoma: results from a radiogenomics study of 111 patients. Neuro-oncology 21 (1), 115–124. 10.1093/neuonc/noy093 29846693 PMC6303469

[B5] Dufour C. Beaugrand A. Pizer B. Micheli J. Aubelle M. S. Fourcade A. (2012). Metastatic medulloblastoma in childhood: chang's classification revisited. Int. J. Surg. Oncol. 2012, 245385. 10.1155/2012/245385 22312539 PMC3265270

[B6] Emura T. Matsui S. Chen H. Y. (2019). compound.Cox: univariate feature selection and compound covariate for predicting survival. Comput. methods programs Biomed. 168, 21–37. 10.1016/j.cmpb.2018.10.020 30527130

[B7] Entz-Werle N. Carli E. D. Ducassou S. Legrain M. Grill J. Medulloblastoma D. C. (2008). What is the role of molecular genetics? Expert Rev. anticancer Ther. 8 (7), 1169–1181. 10.1586/14737140.8.7.1169 18588461

[B8] Franceschi E. Seidel C. Sahm F. Pajtler K. W. Hau P. (2021). How we treat medulloblastoma in adults. ESMO open 6 (4), 100173. 10.1016/j.esmoop.2021.100173 34118771 PMC8207184

[B9] Gajjar A. J. Robinson G. W. (2014). Medulloblastoma-translating discoveries from the bench to the bedside. Nat. Rev. Clin. Oncol. 11 (12), 714–722. 10.1038/nrclinonc.2014.181 25348790

[B10] Guo C. Yao D. Lin X. Huang H. Zhang J. Lin F. (2020). External validation of a nomogram and risk grouping system for predicting individual prognosis of patients with medulloblastoma. Front. Pharmacol. 11, 590348. 10.3389/fphar.2020.590348 33343359 PMC7748109

[B11] Huang P. I. Lin S. C. Lee Y. Y. Ho D. M. Guo W. Y. Chang K. P. (2017). Large cell/anaplastic medulloblastoma is associated with poor prognosis-a retrospective analysis at a single institute. Child's Nerv. Syst. 33 (8), 1285–1294. ChNS : official journal of the International Society for Pediatric Neurosurgery. 10.1007/s00381-017-3435-9 28488086

[B12] Iasonos A. Schrag D. Raj G. V. Panageas K. S. (2008). How to build and interpret a nomogram for cancer prognosis. J. Clin. Oncol. 26 (8), 1364–1370. official journal of the American Society of Clinical Oncology. 10.1200/JCO.2007.12.9791 18323559

[B13] Kwong Y. D. Mehta K. M. Miaskowski C. Zhuo H. Yee K. Jauregui A. (2020). Using best subset regression to identify clinical characteristics and biomarkers associated with sepsis-associated acute kidney injury. Am. J. physiology Ren. physiology 319 (6), F979–f87. 10.1152/ajprenal.00281.2020 PMC7792692 33044866

[B14] Liu H. Sun P. (2022). A nomogram model for predicting prognosis of patients with medulloblastoma. Turk. Neurosurg. 10.5137/1019-5149.JTN.40397-22.3 37309623

[B15] Louis D. N. Ohgaki H. Wiestler O. D. Cavenee W. K. Burger P. C. Jouvet A. (2007). The 2007 WHO classification of tumours of the central nervous system. Acta neuropathol. 114 (2), 97–109. 10.1007/s00401-007-0243-4 17618441 PMC1929165

[B16] McEligot A. J. Poynor V. Sharma R. Panangadan A. (2020). Logistic LASSO regression for dietary intakes and breast cancer. Nutrients 12 (9), 2652. 10.3390/nu12092652 32878103 PMC7551912

[B17] Menyhárt O. Győrffy B. (2020). Molecular stratifications, biomarker candidates and new therapeutic options in current medulloblastoma treatment approaches. Cancer metastasis Rev. 39 (1), 211–233. 10.1007/s10555-020-09854-1 31970590 PMC7098941

[B18] Northcott P. A. Robinson G. W. Kratz C. P. Mabbott D. J. Pomeroy S. L. Clifford S. C. (2019). Medulloblastoma. Nat. Rev. Dis. Prim. 5 (1), 11. 10.1038/s41572-019-0063-6 30765705

[B19] Packer R. J. Gajjar A. Vezina G. Rorke-Adams L. Burger P. C. Robertson P. L. (2006). Phase III study of craniospinal radiation therapy followed by adjuvant chemotherapy for newly diagnosed average-risk medulloblastoma. J. Clin. Oncol. 24 (25), 4202–4208. official journal of the American Society of Clinical Oncology. 10.1200/JCO.2006.06.4980 16943538

[B20] Rieken S. Mohr A. Habermehl D. Welzel T. Lindel K. Witt O. (2011). Outcome and prognostic factors of radiation therapy for medulloblastoma. Int. J. Radiat. Oncol. Biol. Phys. 81 (3), e7–e13. 10.1016/j.ijrobp.2010.12.042 21345611

[B21] Schwalbe E. C. Lindsey J. C. Nakjang S. Crosier S. Smith A. J. Hicks D. (2017). Novel molecular subgroups for clinical classification and outcome prediction in childhood medulloblastoma: a cohort study. Lancet Oncol. 18 (7), 958–971. 10.1016/S1470-2045(17)30243-7 28545823 PMC5489698

[B22] Thompson E. M. Hielscher T. Bouffet E. Remke M. Luu B. Gururangan S. (2016). Prognostic value of medulloblastoma extent of resection after accounting for molecular subgroup: a retrospective integrated clinical and molecular analysis. Lancet Oncol. 17 (4), 484–495. 10.1016/S1470-2045(15)00581-1 26976201 PMC4907853

[B23] Zhu S. Lin F. Chen Z. Jiang X. Zhang J. Yang Q. (2020). Identification of a twelve-gene signature and establishment of a prognostic nomogram predicting overall survival for medulloblastoma. Front. Genet. 11, 563882. 10.3389/fgene.2020.563882 33101383 PMC7495025

