# Supplementary material for: Survival nomogram for medulloblastoma and multi-center external validation cohort
Source: Front Pharmacol. 2023 Nov 2;14:1247812. doi: 10.3389/fphar.2023.1247812 (PMC10651750; doi:10.3389/fphar.2023.1247812)
Supplement: Supplementary file 1 [file Presentation1.pptx]

## Slide 1
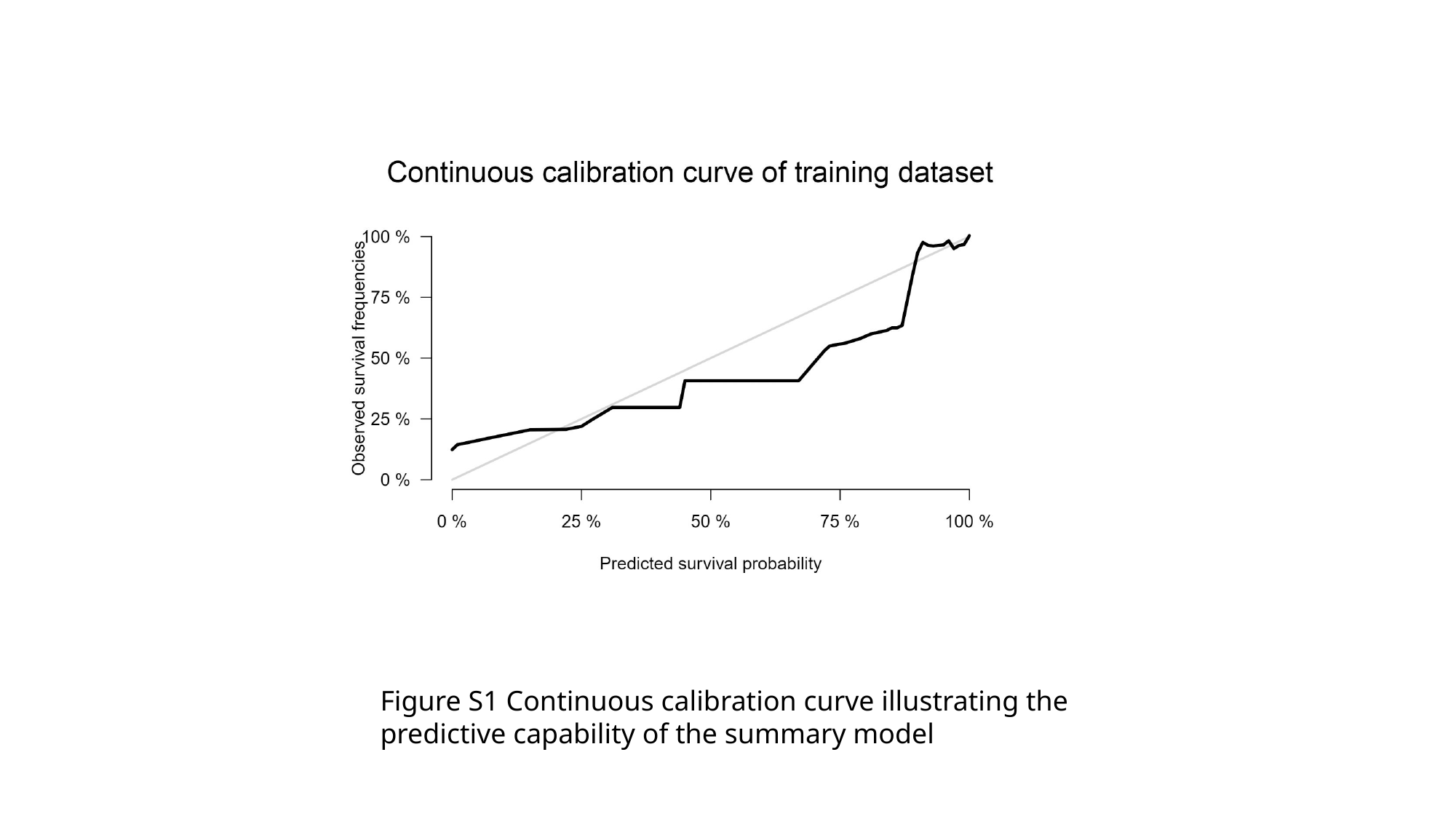

Figure S1 Continuous calibration curve illustrating the predictive capability of the summary model

## Slide 2
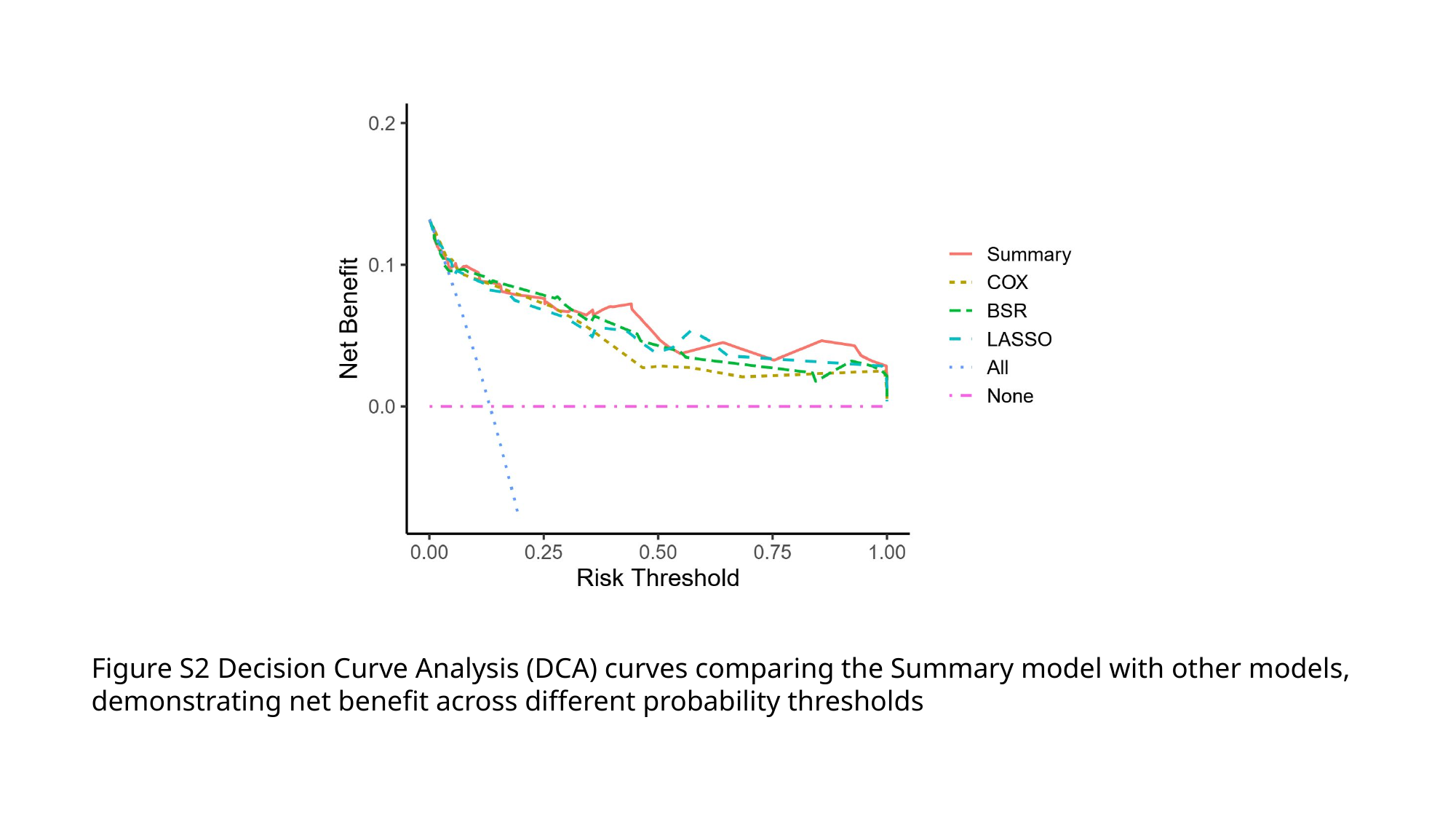

Figure S2 Decision Curve Analysis (DCA) curves comparing the Summary model with other models, demonstrating net benefit across different probability thresholds

## Slide 3
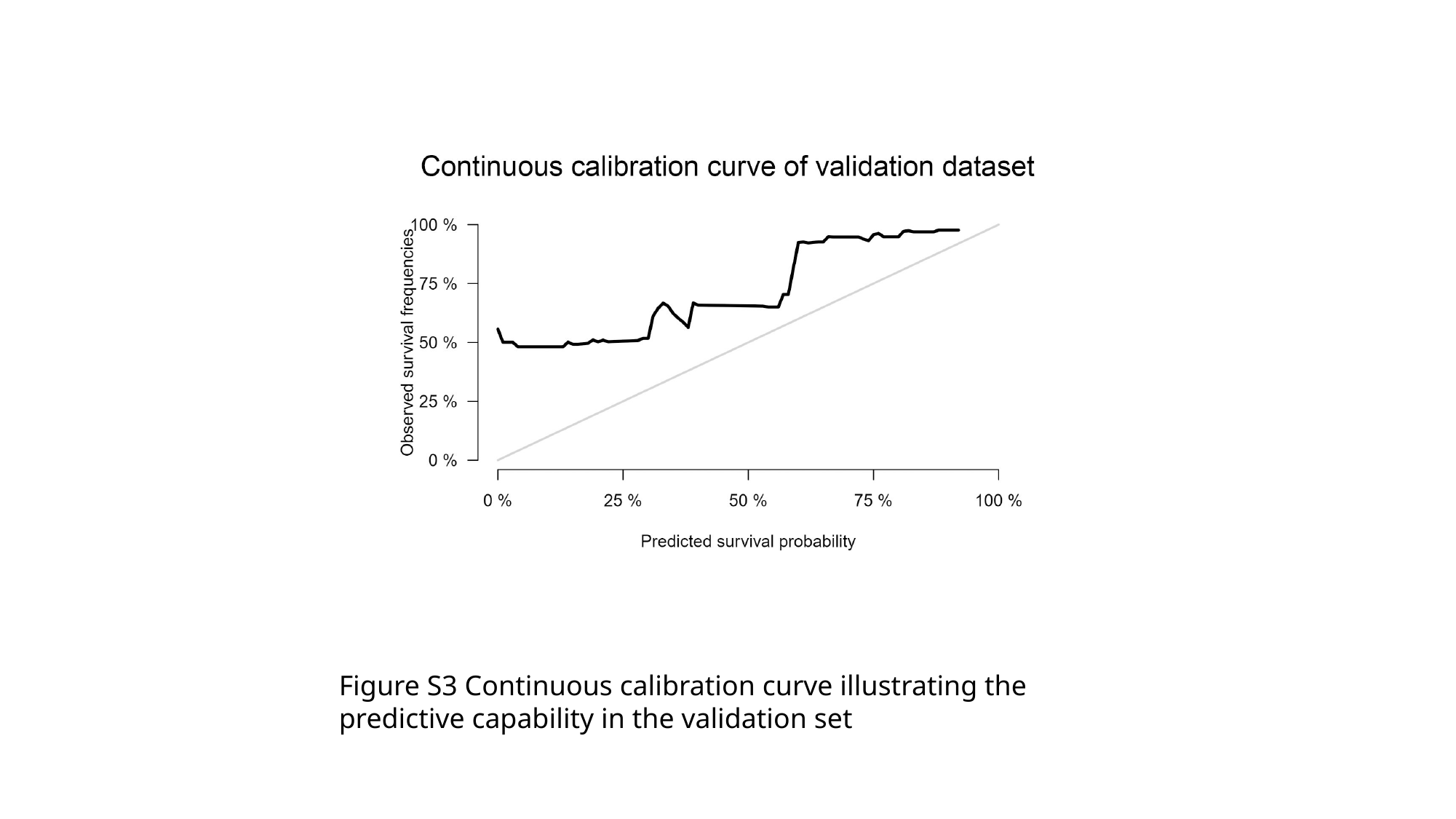

Figure S3 Continuous calibration curve illustrating the predictive capability in the validation set
